# Fractionation of the rice bran layer and quantification of vitamin E, oryzanol, protein, and rice bran saccharide

**DOI:** 10.1186/1754-1611-1-9

**Published:** 2007-12-28

**Authors:** Rebecca Schramm, Alicia Abadie, Na Hua, Zhimin Xu, Marybeth Lima

**Affiliations:** 1 Department of Biological and Agricultural Engineering, Louisiana State University, Baton Rouge, LA 70803, USA; 2 Department of Biosystems and Agricultural Engineering, University of Kentucky, Lexington, KY 40546, USA; 3 Louisiana Department of Health and Hospitals, Baton Rouge, LA 70802, USA; 4 Department of Food Science, LSU AgCenter, Baton Rouge, LA 70803, USA; 5 Department of Biological and Agricultural Engineering, LSU AgCenter, Baton Rouge, LA 70803, USA

## Abstract

Value-added processing with respect to rice milling has traditionally treated the rice bran layer as a homogenous material that contains significant concentrations of high-value components of interest for pharmaceutical and nutraceutical applications. Investigators have shown that high-value components in the rice bran layer vary from differences in kernel-thickness, bran fraction, rice variety, and environmental conditions during the growing season. The objectives of this study were to quantify the amount of rice bran removed at pre-selected milling times and to correlate the amount of rice bran removed at each milling time with the concentration of vitamin E, gamma-oryzanol, rice bran saccharide, and protein obtained. The ultimate goal of this research is to show that rice bran fractionation is a useful method to obtain targeted, nutrient-rich bran samples for value-added processing. Two long grain rice cultivars, Cheniere and Cypress, were milled at discrete times between 3 and 40 seconds using a McGill mill to obtain bran samples for analysis. Results showed that the highest oryzanol and protein concentrations were found in the outer portion of the rice bran layer, while the highest rice bran saccharide concentration was found in the inner portion of the bran layer. Vitamin E concentration showed no significant difference across the bran layer within a variety, though the highest magnitude of concentration occurs within the first 10 seconds of milling for both varieties. To extract the higher concentration of oryzanol and protein only the outer portion of the bran layer requires processing, while to extract the higher concentration of rice bran saccharide, only the inner portion of the bran layer requires processing. Rice bran fractionation allows for the selective use of portions of the bran layer and is advantageous for two reasons: (1) bran fractions contain higher concentrations of components of interest with respect to the overall bran layer average, and (2) less bran needs to be processed to obtain components of interest.

## Background

The importance of rice to the world population's dietary requirement is evident from its presence in the diet of a quarter of the world's people [1]. Rice processing or milling produces several streams of material, including husks, milled rice, and bran. In the United States, rice bran material is considered a by-product of the milling process and is most commonly used in animal feed or as a food ingredient due to its high nutritional content [2].

As interest in value-added processing research grows, attempts are being made to increase the value of agricultural crop by-products, including rice bran, by increasing their pharmaceutical or nutraceutical potential. While rice bran has traditionally been utilized for pet food products [3], there is growing interest in the wide array of potentially human-health enhancing compounds found in rice bran. Rice bran is a good source of antioxidants including vitamin E and oryzanol, high quality oil and protein, and cholesterol-lowering waxes and anti-tumor compounds like rice bran saccharide [4-6]. Value-added processing to obtain these phytochemicals has the potential to be an important way of improving human health while increasing economic rates of return.

In the unsaponifiable portion of rice bran oil, two groups of antioxidant compounds were identified as tocotrienols and gamma (γ)-oryzanol [7]. Tocotrienols, which are members of the vitamin E family, and γ-oryzanol, have been studied for potential health benefits [7]. Epidemiological studies have shown that antioxidants reduce oxidative damage to bimolecular structures that play a role in the prevention of chronic diseases. Antioxidants may help slow the onset of diabetes and Alzheimer's disease, and appear to play a role in the prevention of heart disease and cancer [8]. Tocotrienols have been shown to address free radicals in cell membranes and help in the prevention of coronary artery disease; γ-oryzanol (oryzanol) has been shown to lower blood cholesterol and to reduce levels of cholesterol in the liver [6].

Rice bran saccharide (RBS), a polysaccharide component contained in rice bran, exhibits anti-tumor capabilities [4]. In a study on tumor prevention and suppression of tumor growth in rats, RBS was found to suppress carcinogenesis and to prolong survival rate [9]. We have not seen any published literature regarding the fractionation of the bran layer to examine RBS concentration. If RBS can be extracted successfully, rice bran could be a source for a high-value pharmaceutical product [9].

In studies of rice bran, milling times, kernel-thickness fraction, variety, and environmental conditions have been investigated. Bergman and Xu [10] found variation in the levels of tocopherol, tocotrienol, and γ-oryzanol for southern U.S. rice cultivars, including AB647, Bengal, Cypress, Dellmati, LaGrue, Toro 2, and Wells. Their work indicated that the content of these phytochemicals appeared to be influenced by variety and geographic location. Rohrer and Siebenmorgen [6] divided rough rice into three thickness fractions for rice varieties Cypress and Drew. Bran from thicker kernel fractions contained higher levels of nutraceuticals than bran from thinner kernel fractions. They also found that bran collected from shorter milling times resulted in higher levels of tocopherols and tocotrienols than for longer milling times.

This study was motivated by our observation of different concentrations of high-value compounds between varieties and across the bran layer within varieties during pilot scale milling [11]. These differences were not very pronounced because it was difficult to obtain precise bran fractions on the pilot scale mill. We sought to extend research in this area by testing precise bran layer fractions for a number of high-value compounds contained in the rice bran layer. The objectives of this study were: (1) to quantify the amount of rice bran removed at selected process time settings, and (2) to correlate the amount of rice bran removed at a given process time setting with the concentration of RBS, protein, vitamin E, and γ-oryzanol present.

## Methods

Two long grain rice varieties, Cypress and Cheniere, were milled at the laboratory scale. Bran fractions were collected and tested to determine the amount of bran removed at discrete milling times. Bran fraction was defined as the weight of bran removed at each process time setting (milling time) divided by the weight of the shelled rice processed, and is reported on a percent basis. For each replication, bran samples were properly stabilized and stored [12] until analysis to determine vitamin E, γ-oryzanol, protein, and RBS concentration. Experiments of each variety-milling time-component combination were executed randomly using three repetitions for each combination. After ANOVA was used to establish that differences among means existed using SAS [13], specific differences were determined using the two-tailed Student t-test at the 5% significance level (Microsoft Professional 2003 Excel Data Analysis Tools).

### Milling

Rice was supplied by the LSU Agricultural Center (Louisiana Rice Research Station, Crowley, LA), and remained in cold storage (-18°C) until required for testing. The day before processing, rice was removed from cold storage, allowing time for the rice to equilibrate to ambient temperature. Before milling, the average moisture content of each sack (approximately 23 kilograms) was determined from measurements made with a grain analyzer (Dickey-John, Model GAC II, Auburn, IL). Average initial moisture content was 14.30 ± 0.35%.

Rough rice samples of 175 grams were processed through a shelling unit (McGill Sheller, Model MS1, Brookshire, Texas). After shelling, brown rice samples of 125 grams were milled (McGill mill, Model No. 2, Brookshire, Texas).

For vitamin E and γ-oryzanol analysis, samples of brown rice were milled in triplicate at nine time settings from 5 to 45 seconds in 5-second intervals; streams of milled rice and bran were collected and weighed. Five-gram bran samples were collected for each replicate (54 samples), and heat stabilized using the method described in [13].

Bran samples for protein and RBS analysis were collected from 125 gram brown rice samples processed with the McGill mill for the following times: 3, 5, 10, 20, and 40 seconds. A 3-second milling time was added after initial data analysis of vitamin E and γ-oryzanol showed high concentrations of these compounds at short milling times (<10 seconds). Longer time intervals for longer milling durations were chosen accordingly. At the shorter milling times, multiple 125 gram samples of brown rice were milled until collection of 10 grams of material were obtained for analysis.

### Vitamin E and γ-Oryzanol Analysis

Normal phase and reverse phase high pressure liquid chromatography (HPLC) were used to determine the concentration of vitamin E and γ-oryzanol using the methods detailed in [14] and [15], respectively. Standards were obtained from Sigma-Aldrich. For vitamin E., the mobile phase was hexane, ethyl acetate, and acetic acid (99%, 0.5%, and 0.5%, respectively) and the flow rate was 1.8 ml/min. Excitation and emission wavelengths of fluorescence detector were 290 and 330 nm, respectively. Vitamin E concentration was reported as the sum of concentrations of α-tocopherol, α-tocotrienol, γ-tocopherol and γ-tocotrienol. The reverse phase HPLC system used to measure γ-oryzanol concentration consisted of a C18 column (Rainin Instrument Company, Woburn, MA), auto sampler, absorbance detector, and UV detector. The mobile phase was methanol, acetonitrile, dichloromethane, and acetic acid (50:44:3:3) and the mobile phase flow rate was 2 ml/min. The wavelength of UV detector was 330 nm (Waters, Milford, MA). HPLC analysis of each variety, milling time, and component combination was conducted in triplicate.

### Protein Analysis

Protein was measured with a nitrogen analyzer (Perkin Elmer Series II Nitrogen Analyzer 2410, Sheldon, CT) based on the Dumas method [16]. Samples were combusted, then nitrogen was separated from other product gases by frontal chromatography and quantified by thermal conductivity (Perkin Elmer Series II Nitrogen Analyzer 2410, Online Manual). After calibration of the analyzer, 0.2 gram rice bran samples were processed. Results were automatically reported by the analyzer in weight percent nitrogen, converted to weight percent protein, and reported in mg/g of rice bran.

### RBS Analysis

Quantification of RBS, a polysaccharide, required its removal from the rice bran layer by an enzymatic extraction. The method used was a modified version of Ito et al. [17] and Hanmoungjai et al. [2]. Ito et al. [17] used a one enzyme treatment with α-amylase, and Hanmoungjai et al. [2] used an enzymatic treatment employing several carbohydrases. Thirty milligrams each of two enzymes, cellulase (*Aspergillus niger*, Sigma-Aldrich) and xylanase (*Trichoderma viride*, Sigma-Aldrich), broke down the rice bran matrix prior to extraction to obtain the total concentration of saccharide. A separate non-enzymatic process extracted monosaccharides and oligosaccharides. The two extraction solutions were analyzed for saccharide content with a colorimetric method [18]. Saccharide concentration was quantified using a spectrophotometer (Spectronic 20+, Spectronic Instruments, Waltham, MA) at 490 nm, after calibration with known concentrations of D-(+) glucose (Sigma-Aldrich). RBS concentration was determined from the difference in saccharide content measured in the two extraction solutions.

## Results and Discussion

### Vitamin E and γ-oryzanol

**Figure 1:**
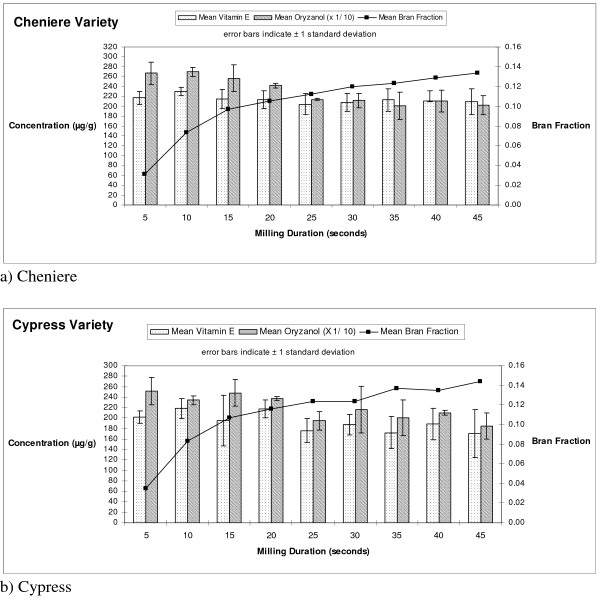
Vitamin E concentration, oryzanol concentration, and percent bran fraction as a function of milling time (p < 0.05).

Table 1 shows the concentration of vitamin E and oryzanol as a function of milling time for Cheniere and Cypress (Figure 1). The concentration of vitamin E is essentially flat across the bran layer as a function of milling time. There is no significant difference in vitamin E concentration among milling times within a variety. While not statistically significant, the highest concentration of vitamin E is present at the 10-second setting for Cypress and Cheniere, and the second highest concentration is obtained at the 5-second setting. No significant difference in vitamin E concentration exists between the varieties at the 5- or 10-second milling times. Differences do exist at other milling times between varieties, including a consistent difference between 15 and 25 seconds of milling.

**Table 1:** Vitamin E and oryzanol concentration and bran fraction as a function of time for Cypress and Cheniere rice varieties (Mean values ± one standard deviation reported)

**Variety:**	**Cypress**			**Cheniere**		
**Time (seconds)**	**Mean Vitamin E (μg/g)**	**Mean Bran Fraction**	**Mean Oryzanol (μg/g)**	**Mean Vitamin E (μg/g)**	**Mean Bran Fraction**	**Mean Oryzanol (μg/g)**

5	202.13 ± 11.71	0.03 ± 0.002	2516.24 ± 255.61	217.02 ± 13.86	0.03 ± 0.002	2671.19 ± 230.98
10	218.21 ± 18.62	0.08 ± 0.008	2338.71 ± 94.21a	229.93 ± 8.39	0.07 ± 0.001	2699.05 ± 90.42a
15	195.02 ± 48.58 a	0.11 ± 0.000	2480.83 ± 264.78	214.75 ± 20.00 a	0.10 ± 0.003	2564.12 ± 273.88
20	217.86 ± 16.94 a	0.12 ± 0.002	2374.48 ± 39.74	213.19 ± 17.63 a	0.11 ± 0.004	2418.41 ± 45.98b10
25	175.73 ± 22.62 a	0.12 ± 0.000	1946.37 ± 180.14b5,10,15,20	204.20 ± 21.81 a	0.11 ± 0.004	2136.72 ± 20.40b5,10,15,20
30	187.58 ± 19.64	0.12 ± 0.008	2161.72 ± 451.80	208.29 ± 18.29	0.12 ± 0.004	2114.77 ± 150.10c
35	172.26 ± 31.46 a	0.14 ± 0.003	2009.12 ± 341.61	212.78 ± 22.58 a	0.12 ± 0.005	2010.36 ± 283.10b5,10
40	188.76 ± 30.01	0.13 ± 0.009	2102.17 ± 43.47b5,10,20	210.66 ± 20.43	0.13 ± 0.004	2110.25 ± 218.93b5,10
45	170.26 ± 45.63 a	0.14 ± 0.001	1846.98 ± 248.32b5,10,20,25	209.12 ± 25.47 a	0.13 ± 0.004	2022.55 ± 186.08b5,10,15,20

a-statistically different between varieties at same operational time setting

b-statistically different within a variety between operational time settings listed

c-one less data point

The highest concentrations of γ-oryzanol are obtained in the outer portion of the bran layer for both varieties of rice. The highest observed concentration of γ-oryzanol occurs at different milling times for Cypress and Cheniere. For Cypress, the highest γ-oryzanol concentrations occurs at the 5 second milling time, and for Cheniere occurs at the 10 second milling time. Within the same variety, significant differences in γ-oryzanol concentration occur between the 20 and 25 second milling times for Cypress and Cheniere, indicating high concentrations of γ-oryzanol in the outer half of the bran layer for each rice variety. In general, there is no significant difference in γ-oryzanol concentration between varieties.

Rohrer and Siebenmorgen (2004) [6] observed that the highest concentration of γ-oryzanol occurred at a 10 second milling time for rice varieties Cypress and Drew. These findings are similar to those of our study, and collectively indicate that the highest concentration of γ-oryzanol is contained in the outer portion of the bran layer for three rice varieties.

Mean values for Cypress of 192.0 μg/g of vitamin E and 2197.40 μg/g of γ-oryzanol were obtained in our study. Bergman and Xu [10] found a mean vitamin E value of 309.37 mg/kg (μg/g) for Cypress and a mean value of 4144.54 mg/kg (μg/g) for oryzanol. Our study resulted in a ratio of vitamin E to γ-oryzanol of 11.4 for Cypress, while [10] obtained a ratio of 13.3. Bergman and Xu [10] used an extraction method without a saponification step; we used an extraction method with a saponification step which lowers extraction recovery by causing hydrolysis of γ-oryzanol.

The amount of bran removed as a function of milling time is reported as a fraction of the total bran removed. A Langmuir trend of increasing bran fraction with increasing process time setting is observed. Cypress and Cheniere exhibit statistical differences in bran removal at some of the milling times (15, 20, 25, 35, 45 seconds). These differences may be explained by variety variances in adhesion of bran to the rice kernel. Within rice variety, differences are observed between successive milling times until the 25 and 30 second milling times (p-values < 0.05). Between successive higher milling times, no significant differences occur.

### Protein and rice bran saccharide

**Figure 2:**
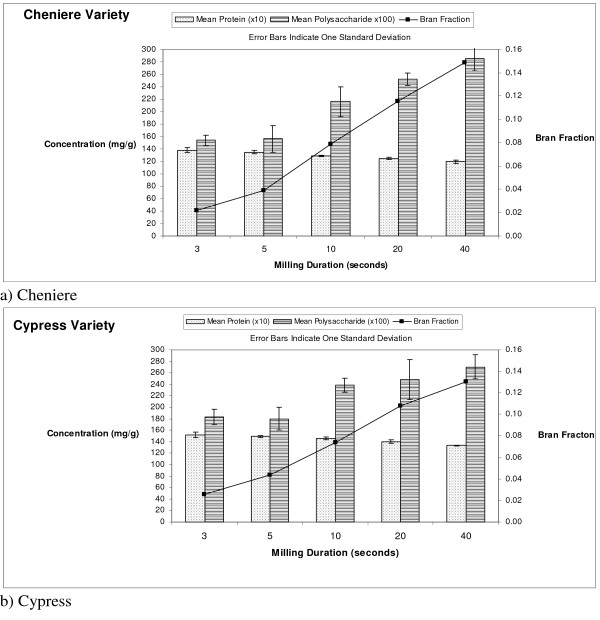
Mean protein, RBS concentration and percent bran fraction as a function of milling time (p < 0.05).

Table 2 contains the results for protein and RBS concentration as a function of milling time for each rice variety (Figure 2). Cypress and Cheniere show decreasing protein concentration with increasing milling time. Within variety analysis shows that a significant difference exists between the 10- and 20-second milling times for Cypress and between the 5- and 10-second milling times for Cheniere (p-values < 0.05). The highest protein concentration occurs at the shorter milling times for both varieties, indicating that the majority of protein is found in the outer portion of the bran layer. Rohrer and Siebenmorgen [6] found that shorter milling times correlate to the removal of the germ and aleurone layer inside the bran; we hypothesize that protein is contained in these portions of the bran layer. Between varieties, Cypress has significantly more protein than Cheniere across the bran layer.

**Table 2:** Protein and RBS Data for Cypress and Cheniere Rice Varieties (Mean values ± one standard deviation reported)

**Variety:**	**Cypress**			**Cheniere**		
**Time (seconds)**	**Mean Protein (mg/g)**	**Mean Bran Fraction**	**Mean RBS concentration (mg/g)**	**Mean Protein (mg/g)**	**Mean Bran Fraction**	**Mean RBS (mg/g)**

3	15.15 ± 0.470 a	0.03 ± 0.001	0.551 ± 0.133 a	13.81 ± 0.382	0.02 ± 0.000	0.300 ± 0.081 a
5	14.89 ± 0.168 a, b3	0.04 ± 0.008	0.532 ± 0.197	13.47 ± 0.246	0.04 ± 0.006	0.510 ± 0.223
10	14.56 ± 0.159 a,b3	0.07 ± 0.013	0.532 ± 0.121	12.93 ± 0.128 b3,5	0.08 ± 0.002	0.459 ± 0.240
20	13.99 ± 0.256 a,b3,5,10	0.11 ± 0.001	0.902 ± 0.352 b3,5	12.49 ± 0.178 b3,5,10	0.12 ± 0.001	0.615 ± 0.098
40	13.32 ± 0.094 a,b3,5,10,20	0.13 ± 0.016	1.148 ± 0.208 b5	11.97 ± 0.339 b3,5,10	0.15 ± 0.001	0.988 ± 0.200 b10,20

a-statistically different between varieties at same operational time setting

b-statistically different within a variety between operational time settings listed

Cypress and Cheniere show increasing rice bran saccharide concentration with increasing milling time, indicating that RBS is concentrated in the inner bran layer. There is no significant difference in RBS concentration between rice varieties at all milling times except 3 seconds, where Cypress has a higher concentration of RBS than Cheniere. Within each variety, statistical differences emerge at different milling times after approximately 20 seconds of milling. Both varieties exhibited the highest measured RBS values at 40 seconds with 1.148 mg/g (1148 mg/kg) for Cypress and 0.988 mg/g (988 mg/kg) for Cheniere. Ito et al. [17] used doses of 10 mg/kg and 100 mg/kg of RBS in animal anti-tumor activity studies with positive tumor reduction results. A kilogram of rice bran from Cypress or Cheniere contains a RBS concentration approximately an order of magnitude higher than the doses used in the animal anti-tumor studies.

### Implication for bran fractionation

**Figure 3:**
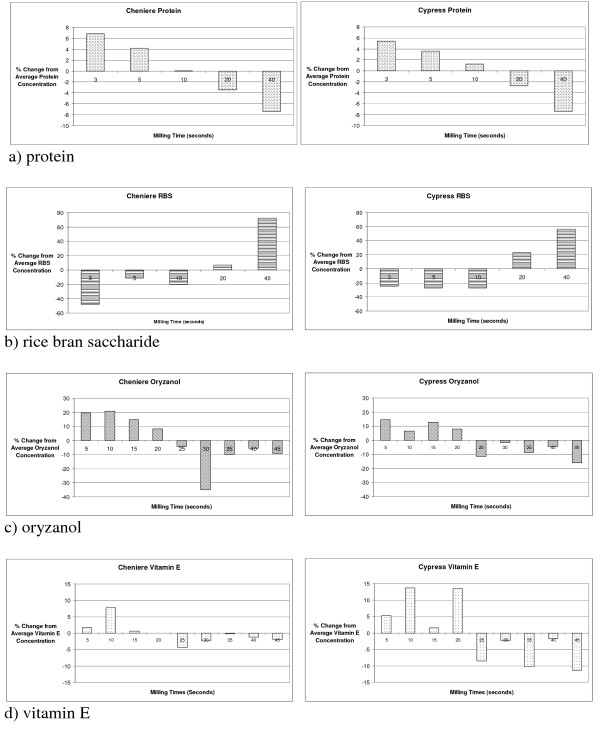
Percent difference in component concentration with respect to the overall component average as a function of milling time.

The high-value compounds in this study exhibited optimal concentration at different locations within the rice bran layer. The following strategy for selective fractionation is thus suggested: vitamin E, oryzanol, and protein should be extracted from the bran fraction collected at or below a 20-second milling time, and RBS should be extracted from the fraction of bran produced above a 20-second milling time. Vitamin E is included in the fraction collected at or below the 20-second milling time based on the location of highest measured concentration. While there is not a significant difference in vitamin E concentration across milling times, both varieties exhibit higher concentration in the 20 seconds of milling. Figure 3 graphically presents the percent change from average values by variety and compound.

A division of the bran at the 20-second milling time would facilitate a value-added approach, as it would require less material to be processed while simultaneously offering an enriched fraction to process. Splitting the bran layer into fractions for value-added processing is potentially transferable to industrial scale milling with two, three, or four break systems.

## Conclusion

Value-added processing with respect to rice milling has traditionally treated the rice bran layer as a homogenous material that contains significant concentrations of high-value components. Rice bran fractionation allows for the selective use of portions of the bran layer and is advantageous for two reasons: (1) bran fractions contain much higher concentrations of components of interest with respect to the overall bran layer average, and (2) less bran needs to be processed to obtain components of interest. This research is important to the biological engineering community because it uses bioprocess engineering methods to isolate health related components of interest in a biological product.

Oryzanol and protein concentrations were found to be the highest in the outer portion of the rice bran layer, while RBS concentration was found to be the highest in the inner portion of the bran layer. Vitamin E concentration was highest at the 10-second milling time, but no significant difference existed between milling times within a variety.

This laboratory scale study illustrates the potential for selectively fractionating the bran layer to obtain small, nutrient rich bran samples. Additional studies involving model development for industrial scale-up are warranted; our hypothesis is that rice bran collected at the first milling break will be oryzanol and protein rich. Future work in this area could include rice variety development and process optimization with a focus on increasing the concentration of high value components, and retaining the bio-activity of high-value components at the industrial scale. Taken collectively, research indicates that milling times, kernel-thickness, bran fraction, rice variety, and environmental conditions can be used to select for high concentrations of specific phytochemicals in rice bran, including several not quantified in this study, e.g. rice bran oil and waxes.

## Authors' contributions

RS conducted experiments on vitamin E and oryzanol, AA conducted experiments on rice bran saccharide, and NH conducted experiments on protein. Additionally, these three co-authors were involved with data analysis. ZX conducted HPLC analysis (as did NH). ML conceived of this study and participated in its design and coordination. RS, AA, and ML wrote the manuscript. All authors approved the final manuscript.
